# Subacute Degeneration of Fibers After Vertical Parasagittal Hemispherotomy

**DOI:** 10.1007/s00062-024-01427-x

**Published:** 2024-06-25

**Authors:** Markus Hittinger, Till Hartlieb, Dieter Henrik Heiland, Pamela Heiland, Tom Pieper, Martin Staudt, Ansgar Berlis, Manfred Kudernatsch, Irina Mader

**Affiliations:** 1 Specialist Centre for Radiology, Schoen Clinic Vogtareuth, Vogtareuth, Germany; 2 Clinic for Diagnostic and Interventional Radiology and Neuroradiology, University Medical Centre, Augsburg, Germany; 3 Clinic for Neurosurgery, University Medical Centre, Freiburg, Germany; 4 Specialist Centre for Paediatric Neurology, Neurorehabilitation and Epileptology, Schoen Clinic Vogtareuth, Vogtareuth, Germany; 5 Specialist Centre for Neurosurgery and Epilepsy Surgery, Schoen Clinic Vogtareuth, Vogtareuth, Germany; 6 University Children’s Hospital, Basel, Switzerland; 7 Centre for Paediatric Palliative Care, Ludwig Maximilian University of Munich, Munich, Germany; 8 Department of Neurosurgery, Medical Centre—University of Freiburg, Freiburg im Breisgau, Germany; 9 Department of Neurosurgery, University Hospital Erlangen, Erlangen, Germany

**Keywords:** Vertical parasagittal hemispherotomy, Diffusion restriction, Wallerian degeneration, Preoperative hemispheric lesions

## Abstract

**Purpose:**

After vertical parasagittal hemispherotomy a restricted diffusion is often seen ipsilaterally and even distant from the adjacent resection margin. This retrospective cohort study analyses the anatomic site and the time course of the diffusion restriction after vertical parasagittal hemispherotomy.

**Methods:**

Fifty-nine patients were included into this study, all of them having had one pre-operative and at least one post-operative MRI, including diffusion imaging at b‑values of 0 and 1000 s/mm^2^ with a calculated ADC.

**Results:**

Diffusion restriction occurred exclusively on the operated site in all patients. In the basal ganglia, diffusion restriction was present in 37 of 38 patients at the first postoperative day with a duration of 38 days. In the midbrain, the posterior limb of the internal capsule and the thalamus, a restricted diffusion became postoperatively prominent at day 9 in all three localizations, with a duration of 36, 34 and 36 days, respectively. The incidence of thalamic lesions was lower if a preoperative damage had occurred.

**Conclusion:**

The restricted diffusion in the basal ganglia resembles direct effects of the operation at its edges, whereas the later appearing diffusion restriction in the midbrain and the posterior limb of the internal capsule rather belong to a degeneration of the descending fibers being transected by the hemispherotomy in the sense of a Wallerian degeneration. The presence of preoperative hemispheric lesions influences the development of diffusion restriction at subacute fiber degeneration.

## Introduction

Vertical parasagittal hemispherotomy is an operation technique in epilepsy surgery providing a complete disconnection of one cerebral hemisphere without creating a cavity [1]. It was introduced by Delalande in 1992 [2] and evaluated in 2007 [3] showing an early complication-free postoperative course in 94% of patients and a low rate (15%) of late complications such as hydrocephalus.

Eligible patients present with drug-resistant epilepsy and have an underlying structural widespread and—based on neurophysiological investigations and imaging findings—unilateral pathology. Recommended indications are: Congenital hemiplegia from a prenatal vascular insult or haemorrhage, Sturge-Weber syndrome, hemimegalencephaly, complex malformation of one hemisphere, Rasmussen encephalitis, hemiconvulsion-hemiplegia-epilepsy syndrome, sequelae of trauma or infection. Although hemispherotomy will result in contralateral hemiplegia and hemianopia, this disadvantage is reduced in patients that are already preoperatively neurologically affected. Corresponding multifocal and widespread unilateral findings must be present in the EEG, and the contralateral hemisphere must not be visibly affected [1].

**Fig. 1:**
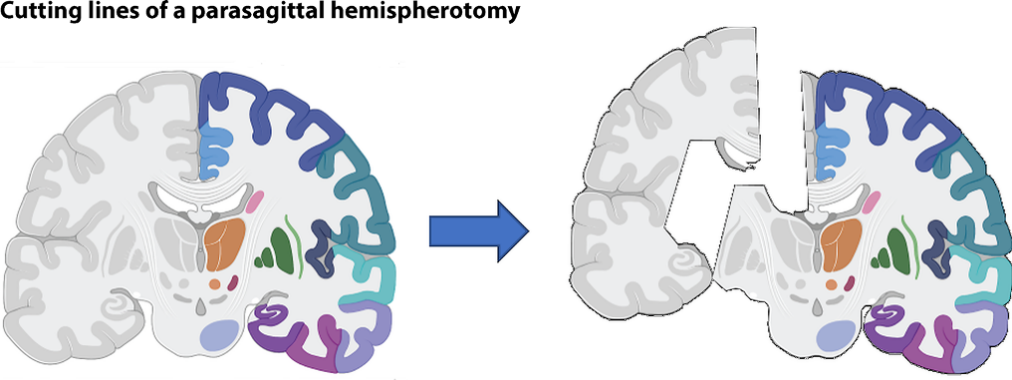
Schematic drawing demonstrating the cutting lines of a parasagittal hemispherotomy; parasagittal hemispherotomy results in a complete disconnection of frontal, parietal, temporal and occipital fibers of the affected hemisphere

Technically, vertical parasagittal hemispherotomy is achieved over a small parasagittal frontal craniotomy. A small cortical resection is performed until the lateral ventricle is reached. After opening of the ventricle and identification of foramen of Monro and thalamus, body and splenium of the corpus callosum are disconnected. The hippocampus becomes disconnected by cutting the posterior column of the fornix at the level of the ventricular trigone. Then a vertical incision is done lateral to the thalamus, between basal ganglia and the insula. It extends from the trigone to the most anterior-mesial part of the temporal horn of the ventricle. Afterwards the completion of the callosotomy is achieved anteriorly and the rostrum of the corpus callosum is disconnected followed by a resection of the posterior part of the gyrus rectus. Then the disconnection is oriented laterally towards the anterior part of the lateral incision (temporal horn). This last step will cut all the connections from the frontal lobe, the anterior temporal lobe and the amygdala ([1]; Fig. 1).

Preoperative high resolution magnetic resonance imaging is necessary to identify the unilateral presence of lesions affecting the whole hemisphere or at least 3 lobes and to confirm that the contralateral hemisphere is not visibly affected. Postoperative imaging is performed to rule out complications such as haemorrhage or hydrocephalus. During the postoperative course, diffusion restriction is often present along the resection edges, but also distant to them in the midbrain, posterior limb of the internal capsule and thalamus. This may be confused with sequelae of vasospasm, although the contralateral vascular territories are not affected which is often the case in vasospasm.

The aim of this study was to retrospectively analyse the anatomic sites and the time course of the diffusion restriction after vertical parasagittal hemispherotomy and to make considerations about the pathomechanism.

## Methods

This study is a retrospective observational cohort analysis of 59 patients having received a parasagittal vertical hemispherotomy between 2012 and 2018 in our children’s epilepsy centre. On admission, the parents or the legal representatives gave written informed consent for the retrospective observational study. Inclusion criteria were: (1) at least one MRI protocol containing diffusion weighted imaging at b‑values of 0 and 1000 s/mm^2^ close to the time of the operation, and (2) at least one post-operative MRI protocol containing diffusion weighted imaging at b‑values of 0 and 1000 s/mm^2^ with a calculated ADC each.

### Patients

Fifty-nine patients (27 females) were included into this study with a median age at operation of 5.24 years (range: 0.6–19.75 years). Duration of epilepsy prior to operation was 3.25 years (median; range: 0.59–19.74 years). The diagnoses included perinatal infarction in 17 patients, complex malformation of one hemisphere in 11 patients, perinatal intraventricular haemorrhage in 8 patients, hemimegalencephaly in 8 patients, polymicrogyria in 6 patients, Rasmussen encephalitis in 5 patients, Sturge-Weber-Syndrome in 2 patients, sequelae of infection in 3 patients, 1 patient after a complex treatment of a brain tumour, mild malformation of cortical dysplasia with oligodendroglial hyperplasia (MOGHE) in 1 patient and tuberous sclerosis in 1 patient. Four patients had two overlapping diagnoses. The outcome according to the Engel Epilepsy Surgery Outcome Scale [4] after two years was: Engel I A in 53 cases, III A in 5 cases, and IV A in 1 case. Preoperative hydrocephalus was present in 2 cases. For complete clinical description please see Table 1.

**Table 1:** Overview of the selected patients including the underlying diagnoses, patients’ ages, duration of epilepsy at the time of the operation, postoperative results and complications

Study population							
Patient numbers	Diagnosis	Sex	Age at operation (years, (Mean and Range))	Duration of Epilepsy (years, (Mean and Range))	Outcome after 2 years (Engel Classification)	Complications	Additional Findings
5	Calcifying tumourous neuroepithelial lesion	M: 1	8.39	7.89	3a	–	–
7, 8, 11, 15, 17, 18, 22, 37, 44, 50, 57	Complex hemispheric cortical dysplasia	F:4 M:7	3.33 (1.21–6.11)	3.04(1.20–6.61)	9: 1a 2: 3a	Hydrocephalus: 3	–
4, 12, 19, 20, 26, 29, 31, 32, 36, 41, 46, 47, 48, 49, 52, 54, 59	Congenital hemiplegia from prenatal vascular insult	F:8 M:9	7.51 (1.87–19.74)	5.77 (0.85–19.74)	16: 1a 1: 4a (case 29)	Hydrocephalus: 2	Col4A1 mutation in case 29
2, 21, 27, 28, 39	Hemimegalencephaly	F:3 M:2	5.60 (0.60–17.05)	4.59 (0.59–13.05)	5: 1a	Hydrocephalus: 1	–
24, 35	Hemimegalencephaly, Polymicrogyria	M:2	10.14 (2.79–17.48)	9.46 (2.29–16.64)	2: 1a	Epidural haemorrhage at day 13 in case 24	–
42	Hemimegalencephaly, Tuberous Sclerosis	F:1	0.87	0.87	1a	–	–
3	Mild malformation of cortical dysplasia with oligodendroglial hyperplasia	M:1	5.39	4.97	1a	–	–
13, 14, 25, 30, 38, 43, 45, 51	Perinatal intraventricular haemorrhage	F: 5 M: 3	8.54 (2.49–19.11)	5.99 (0.83–15.11)	6: 1a 2: 3a	Hydrocephalus: 2	Preoperative CSF shunt in cases 14 and 25, Col4A2 mutation in case 51
33, 34, 40	Polymicrogyria	F: 2 M: 1	7.20 (2.40–11.06)	5.12 (1.68–10.01)	3: 1a	–	–
9, 10, 16, 23, 56	Rasmussen encephalitis	F: 3 M: 2	7.55 (3.68–12.22)	3.10 (1.10–6.66)	5: 1a	Hydrocephalus: 2	–
53, 58	Sequelae of trauma or infection	F: 1 M: 1	6.52 (4.34–8.67)	2.27 (1.59–2.94)	2: 1a	–	–
1, 6	Sturge-Weber Syndrome	M: 2	2.73 (1.67–3.79)	1.99 (0.70–3.29)	2: 1a	–	–
55	Tumor and sequelae of treatment	M: 1	5.23	4.75	1a	Hydrocephalus	Previous treatment of anaplastic astrocytoma

### MRI Protocol

Preoperative MRI consisted of T2- and FLAIR-weighted imaging, either as isotropic 3D-sequences (0.6 mm^3^, 1.2 mm^3^, respectively) or as 2D-sequences (0.45 × 0.45 × 2 mm^3^), and isotropic 3D T1-weighted sequences (1 mm^3^) before and after administration of 0.1 mmol/kg body weight of gadoterate meglumine (Guerbet, Sulzbach, Germany) under general anaesthesia. Preoperative diffusion imaging was performed at 2 b-values (0 and 1000 s/mm^2^) and 12 diffusion directions with a voxel size of 1.8 × 1.8 × 2.4 mm^3^, primarily being used for preoperative fiber tracking, and a map of the trace and the Apparent Diffusion Coefficient (ADC) were calculated.

### Postoperative MRI

At least one postoperative MRI was performed using a short duration protocol in the awake patient with a parent holding the patient within the MR scanner. The protocol contained T2-weighted TrueFISP sequences in transversal (4 mm slice thickness), coronal (4 mm) and sagittal orientation (2 mm), a FLAIR-weighted BLADE-sequence (4 mm slice thickness), a 3D T1-weighted VIBE-sequence (1 mm^3^ voxel size), and a diffusion weighted sequence at 2 b-values (0 and 1000 s/mm^2^) and 3 diffusion directions (5 mm slice thickness) including a calculation of the ADC in each case.

### Data Analysis

Primarily, the preoperative diffusion trace data and the postoperative b‑1000 images of diffusion imaging were co-registered by using the commercially available Syngo MR Neuro 3D Engine (XA20, Siemens, Erlangen, Germany), but postoperative brain shift was severe in all cases, and a manual correction was necessary ending in a co-registration far away from being perfect. So, we choose a mere visual qualitative evaluation. We evaluated the ADC-maps in concordance with the b = 1000 s/mm^2^ images, and rated the ADC as follows: 1 = restricted diffusion, 2 = normal 3 = facilitated diffusion. Rating was performed as consensus reading of two neuroradiologists (MH, 8 years of experience, IM, 29 years of experience). As regions of interest, the basal ganglia were chosen to represent all other structures along the resection site. Furthermore, the peduncle of the midbrain, the posterior limb of the internal capsule and the thalamus were chosen, because these regions developed a diffusion restriction during the evaluation. All regions of interest were on the operated side. The contralateral side was always normal during the postoperative course.

### Statistics

As only a rating on a three-level scale (ranging from 1 (restricted diffusion)–2 (normal)–3 (facilitated diffusion)) was performed, statistics were limited. So, we performed a calculation of the probability by using a Chi-square statistics, with which the diffusion restriction occurred at each day during follow-up.

Statistics was performed by using R (The R Foundation for Statistical Computing, Vienna, Austria, © 2004–2016) and the RStudio (Version 1.2.1335, RStudio, Inc., © 2009–2019).

## Results

On *preoperative imaging* a restricted diffusion could not be found in any localization. Basal ganglia were normal in 54 cases and showed a facilitated ADC in 5 cases. As 25 cases suffered from a partial destruction of the affected hemisphere, e.g. by perinatal stroke or haemorrhage, it was possible to see a facilitated diffusion even prior to the operations. In the midbrain, all 59 cases showed a normal diffusion. In the posterior limb of the internal capsule, 42 cases showed a normal and 17 cases a facilitated diffusion. The thalamus was normal in 42 cases and showed a facilitated diffusion in 17 cases.

**Fig. 2:**
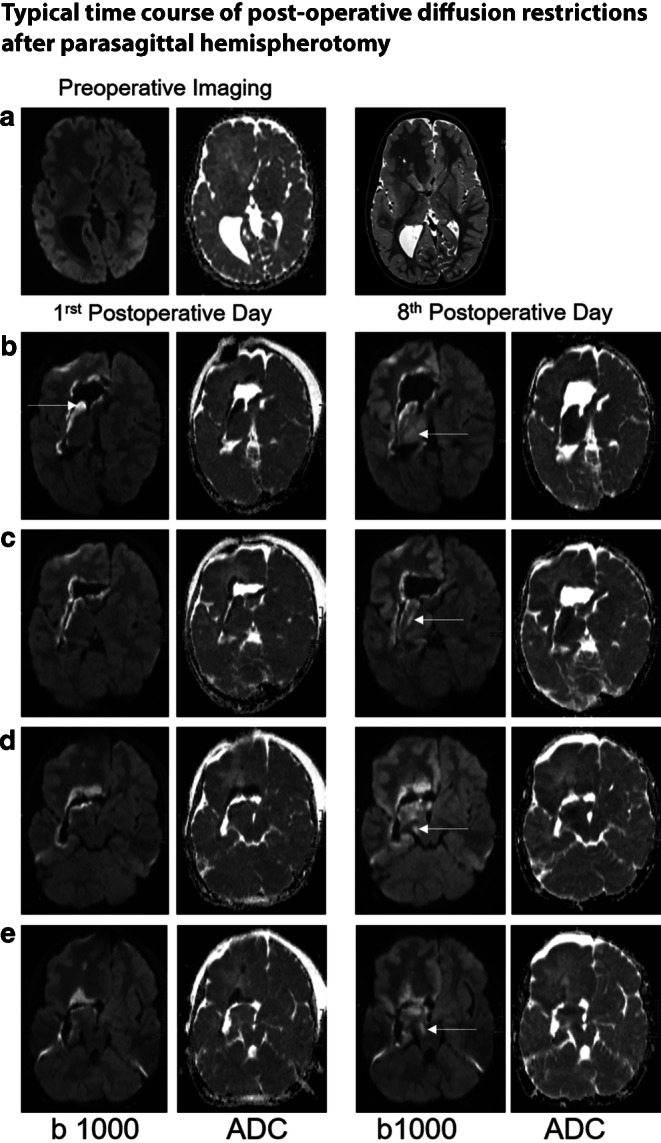
Typical time course of diffusion restrictions after parasagittal hemispherotomy, exemplarily shown on a nearly six years old female patient suffering from a right-sided hemimegalencephaly: The first postoperative MRI investigation (first day after parasagittal hemispherotomy) demonstrates diffusion restrictions that are solely confined to the resection edges. In contrast, the follow-up investigation on the eighth postoperative day shows additional diffusion restrictions within the ipsilateral thalamus (**b**), the ipsilateral posterior limb of the internal capsule (PLIC; **c**) and the ipsilateral cerebral peduncle (**d**, **e**). Within the cerebral peduncle, the occipito-ponto-cerebellar tract (**d**) and the parieto-ponto-cerebellar tract (**e**) are affected [5]

On the first postoperative day, 38 MRI’s were obtained. From days 2 to 38, MR imaging was performed in 125 cases with a median of 2 (range: 0–11) per day. In fact, only on postoperative days 22 and 31 no MRI was performed. From days 39 to 1815 (i.e. 4.97 years after the operation), another 141 MRI’s were obtained. As after day 39 no diffusion restriction was present anymore, investigations after this time point were not subjected to further analysis. A typical time course is given in Fig. 2, showing patient number #39 with a hemimegalencephaly on the right side.

Postoperatively, one epidural haemorrhage occurred on day 13 in patient #24 with a right-sided hemimegalencephaly. An external ventricular drainage was inserted into an isolated trigonum on the right side resulting in an occipital epidural haematoma. After operative revision, the further clinical course was uneventful. Hydrocephalus with the need for implantation of a shunt system occurred in 11 cases (19%; 3 cases of complex hemispherical cortical dysplasia, 2 cases of Rasmussen encephalitis, 2 perinatal intraventricular haemorrhages, 2 perinatal vascular insults, 1 hemimegalencephaly, 1 case after a complex treatment of a brain tumour). A preoperative shunt system was present in 2 cases after prior perinatal intraventricular haemorrhage without further complications (#14, #25). Two other cases, one (#29) with a Col4A1 and one (#51) with a Col4A2 mutation, did not suffer from any complications.

### Time Course of Diffusion Restriction

**Fig. 3:**
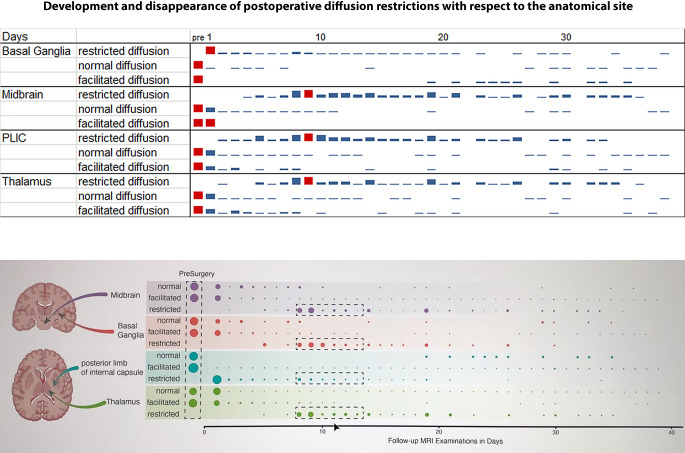
Summary of the development and disappearance of postoperative diffusion restrictions with respect to the anatomical site. Postoperative diffusion restrictions first develop at the resection edges, typically within the first 24 h. The appearance of diffusion restrictions at distant sites (i.e. posterior limb of the internal capsule (PLIC), thalamus and midbrain) is delayed and reaches its maximum after 9 days. Diffusion restrictions disappeared after 38 days at the resection edges, after 36 days within the thalamus and the midbrain and after 34 days within the PLIC

The *basal ganglia *were the only region with a diffusion restriction on the first postoperative day in 37 cases (63%) and a normal diffusion in only 1 case (2%) in 38 MRI’s performed at the first postoperative day. The drop of numbers of diffusion restriction during follow-up is attributed to the fact that from day 2 to 38 only a small number of cases was daily assessed by MRI. Although the maximum of diffusion restriction in the basal ganglia was on day 1, it lasted until day 38 (Fig. 3).

In the *midbrain,* diffusion restriction did not occur during the first three postoperative days. A maximum of diffusion restriction was reached on day 9 (7 cases), compared to the number of cases with normal (1 case) and facilitated restriction (0 cases). Diffusion restriction lasted until day 36 (Fig. 3).

In the *posterior limb of the internal capsule,* diffusion restriction did not occur on the first postoperative day, where diffusion was normal in 33 cases (56%) and facilitated in 5 cases (8%). A maximum of cases with diffusion restriction was reached at day 9 (*n* = 6), compared to the number of cases with normal (*n* = 2) and facilitated diffusion (*n* = 0). Diffusion restriction lasted until day 34 (Fig. 3).

Also, in the *thalamus*, there was no diffusion restriction on the first postoperative day, where diffusion was normal in 28 cases (47%) and facilitated in 10 cases (17%). A maximum of diffusion restriction was reached on day 9 (7 cases), compared to the number of cases with normal (1 case) and facilitated diffusion (0 cases). Diffusion restriction lasted until day 36 (Fig. 3).

Each diffusion restriction resolved over time, most of them without persisting signal abnormalities, and some of them with facilitated diffusion (10 patients in basal ganglia, 1 in midbrain, 11 in posterior limb of the internal capsule and 18 in thalamus) after day 39.

### Fibers Affected by Diffusion Restriction

Between day 5 and 18, within the peduncle of the midbrain the temporopontocerebellar, the occipitopontocerebellar, the parietopontocerebellar tract and the pyramidal tract showed a diffusion restriction, whereas the frontopontocereballar tract was not affected. The assignments were done according to Kamali et al. (2010) [5]. Diffusion restriction occurred in 14/27 cases without preoperative damage of the fibers, in 5/10 cases with a prior damage of the descending motor fibers alone, and in 10/22 cases with prior damage of the descending motor fibers and the optic system (Fig. 3).

During the same period, descending fibers within the posterior limb of the internal capsule revealed a diffusion restriction in 10/27 cases without prior fiber damage, in 2/10 cases with prior damage of the descending motor fibers alone, and in 4/22 cases with a prior damage of the descending motor fibers and of the optic system. The diffusion restriction was located in the lateral posterior limb of the internal capsule attributed to corticofugal fibers ([6]; Fig. 3).

The thalamus also revealed different patterns of diffusion restriction in those three groups. In the group without pre-damaged fibers, 20/27 cases (74%) had a diffusion restriction, whereas in the group with pre-damaged descending motor fibers alone 3/10 cases (30%) revealed a diffusion restriction. In the group with a combined previous damage of descending motor fibers and optic system, only 4/20 cases (20%) had a diffusion restriction (Fig. 3).

### Diffusion Restriction and Vessel Territories

Diffusion restriction occurred in different vessel territories (direct branches of the posterior cerebral artery, thalamostriate arteries and anterior choroid artery) which is not compatible with a territorial stroke. Intracranial arteries had been patent in all cases before the operations. The T1-hyperintense inflow signal of the arteries on the VIBE-sequence was postoperatively well present in all cases. The amount of blood degradation products was low and laid predominantly in the temporomesial resection area being the deepest point during the operations.

## Discussion

Our main findings were diffusion restrictions after sagittal hemispherotomy with two different time courses, one having a maximum at the first postoperative day, and the other with a maximum on postoperative day 9. The basal ganglia showed an early maximum on the first postoperative day. Areas with a later maximum of diffusion restriction (day 9) were the peduncle of the midbrain, the posterior limb of the internal capsule and the thalamus, areas being distant to the resection edge.

### Early Diffusion Restriction

In the basal ganglia, the transient diffusion restriction started at the first postoperative day with a maximum and resolved after day 38. Postoperative diffusion restriction with transient duration has been described in 2005 after operation of gliomas [7], and in 2006 after epilepsy surgery [8]. They were discussed as transient changes of the membranous consistence, of cellularity or water content in different tissue compartments. After resolution of the diffusion restriction, they were replaced by encephalomalacia, a finding being also present in our patients. A resolution of the diffusion restriction occurred after the 50th postoperative day [7], or from the 7th postoperative day up to one month [8]. So, our results along the resection site are in line with the literature.

### Subacute Diffusion Restriction

In the midbrain, the posterior limb of the internal capsule and the thalamus, another time course was found with a maximum at postoperative day 9 that resolved after day 36. Distant changes of diffusivities and anisotropy after dissection of neural tissue have been described in different animal studies for a better understanding of Wallerian degeneration. Changes of diffusion were histologically accompanied by a significant loss of axons at day 3 and a severe loss of axons and myelin at day 14. The changes persisted up to day 30 and were accompanied by swollen axons and formation of myelin ovoids [9–12]. In conclusion, the changes of the ADC in our patient cohort, being anatomically distant to the lesion and timely distant to the operation, are in line with the literature investigating axonal destruction in animal models.

In humans, Wallerian degeneration of the pyramidal tract in the cerebral peduncle was described by diffusion tensor imaging after stroke in the territory of the middle cerebral artery in adults. Within 2–16 days, the longest eigenvalue was reduced, and the smallest eigenvalue was elevated. Whereas no clear changes of averaged diffusivity were present in the subacute stage, in the chronic stage (up to day 288) an increase became prominent [9]. A reduced ADC of descending fibers in the cerebral peduncle after stroke has only rarely been described in adults [10], but was more frequently reported in the cerebral peduncle [11] or in the internal capsule [12] of children, and has been interpreted as an indicator for an early Wallerian degeneration. In neonatal arterial stroke, ADC was decreased along the corticospinal tract within the first 28 days of stroke, in the posterior limb of the internal capsule, the cerebral peduncle, the basis pontis and the medullary pyramids [13].

### Anatomical Considerations Regarding the Affected Fibers

At the level of the midbrain, diffusion restriction occurred in the temporo-ponto-cerebellar, the occipito-ponto-cerebellar (*e.g.*, Fig. 2d), the parieto-ponto-cerebellar (*e.g.*, Fig. 2e) and pyramidal tract [assignment of fibres according to [5]]. Out of all 59 cases, 29 (49%) developed a diffusion restriction at the level of the peduncle of the midbrain. This finding was independent of a prior damage of the fibers. It is not clear why the fronto-ponto-cerebellar fibers are relatively spared compared to the other descending fibers. Every diffusion restriction occurred solely in descending and not in ascending fibers.

In the posterior limb of the internal capsule, only the lateral part containing the corticofugal fibers showed a diffusion restriction [6]. This is an indicator that only descending fibers developed the diffusion restriction, whereas in the medial part of the internal capsule (location of the thalamocortical fibers) no diffusion restriction occurred. In the group without prior damage of the fibers 10/27 cases (37%) developed a diffusion restriction, whereas only 3/32 cases (9%) revealed a diffusion restriction in the group with previous damage. This means that after hemispheral damage, there are only few fibers in the posterior limb of the internal capsule which can develop a diffusion restriction.

There is a similar finding in the thalamus: 20/27 cases (74%) without previous fiber damage developed a diffusion restriction, in contrast to 7/32 cases (22%) with previous fiber damage which experienced a diffusion restriction. This means that the thalamus experienced a reduction of fibers after previous damage. An assignment to single thalamic nuclei is limited by the poor resolution of the diffusion images within our short protocol, but the pulvinar, the mediodorsal nucleus and the ventral lateral anterior and posterior nucleus were affected in both groups [14].

There are clear limitations of this study based on the retrospective design. This leads to small numbers of postoperative MRI on each particular day (median 2 days (range 0–11 days)) and makes it difficult for a statistical evaluation. We might have missed some diffusion restrictions in cases having been not investigated during the first three weeks, and we might have a bias by having investigated more patients with other clinical reasons to perform a control MRI. Indeed, a prospective study design with a follow-up at particular time points would be favourable. However, knowing about a specific progression of changes in the histology after fibre transection in the first 16 days [9–12] allows to draw conclusions about an underlying process from individual examinations at different examination times, especially since most of the examinations took place in the first 21 days. Another problem is the very limited spatial resolution of the postoperative short MRI protocol so that a reliable co-registration of the data sets was not possible. Thus, the assignment of affected fibers is derived purely from anatomical considerations as explained in the text above. Imaging with a higher spatial resolution could have facilitated spatial coregistration, but postoperative brain shift is a problem that also could not be easily solved.

Clinically, our results are in accordance with the literature with a rate of shunt-requiring hydrocephalus of 19%. This is in line with a recent meta-analysis [15] with an incidence of 0–19% including early and delayed postoperative hydrocephalus. There was only one single occipital haemorrhage being not directly attributed to the operative procedure of the hemispherotomy which occurred during the insertion of a shunt catheter 13 days later. Compared to the rate of haemorrhages reported in [15] (10–36%), this is an extremely rare complication in our cohort. Postoperative infections did not occur in our patients. The postoperative outcome after 2 years with Engel I A in 53/59 patients (90%), Engel III A in 5/59 cases (8%), Engel IV A in 1/59 cases (2%) and a mortality of 0% is also in accordance with the literature [1].

## Conclusion

Subacute diffusion restriction distant to the resection edge after vertical parasagittal hemispherotomy with a maximum at day 9 is associated with a degeneration of descending fibers. The severity of the diffusion restriction in the thalamus and the posterior limb of the internal capsule is dependent on their previous damage and more pronounced in cases without previous damage.
